# *Listeria monocytogenes* isolated from a human case of invasive listeriosis survived more than 25 years in a blood culture bottle

**DOI:** 10.1186/s13028-026-00867-4

**Published:** 2026-06-29

**Authors:** Wilhelm Tham, Hannu Korkeala, Gloria Lopez Valladares, Marie-Louise Danielsson-Tham

**Affiliations:** 1 School of Hospitality, Culinary Arts and Meal Science, University of Örebro, Campus Grythyttan, 712 60 Grythyttan, Örebro, Sweden; 2 Department of Food Hygiene and Environmental Health, Faculty of Veterinary Medicine, University of Helsinki, P.O. Box 66, 00014 Helsinki, Finland

**Keywords:** *Listeria monocytogenes*, Persistence, Pulsed-field gel electrophoresis

## Abstract

The aim of the present study was to determine whether *L. monocytogenes* bacteria from an invasive case of listeriosis cultured in a BacT/ALERT FA bottle in 1999 have survived, and if so, whether their pulsed-field gel electrophoresis (PFGE) profiles have changed. In 2024, after more than 25 years in a refrigerator, *L. monocytogenes* was isolated in pure culture from the BacT/ALERT FA bottle. All 100 selected isolates showed the same by PFGE profile with the restriction enzyme *Asc* I as the *L. monocytogenes* isolates did in 1999. *L. monocytogenes* can apparently survive for a long time in a nutrient-poor environment, and this must be taken into account by the food industry. Persistence in clinical samples has rarely been reported; however, several authors report persistence of *L. monocytogenes* in the food industry, with bacteria ending up in final products.

## Findings

**Fig. 1:**
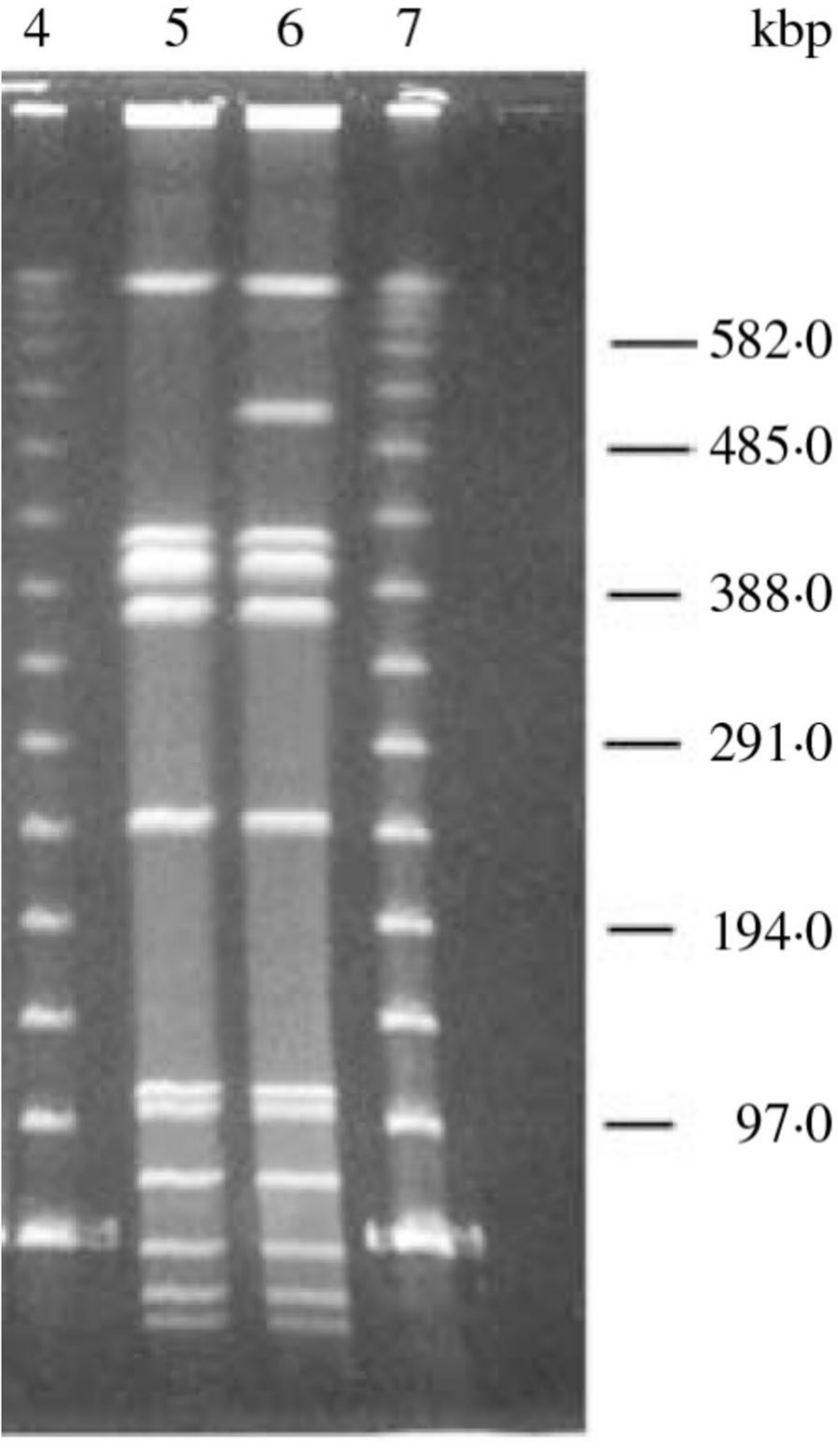
*L. monocytogenes* variants 1 and 2 displayed a two band difference in DNA profile with pulsed-field gel electrophoresis and restriction enzyme *Asc* I. Lanes 4 and 7: Lamda ladder PFG marker (New England BioLabs Inc., USA); lane 5, variant 1; lane 6, variant 2. *Note* The thick band slightly above the 388 kbp band of variant 1 was separated into two bands when the gel was run for another 24 h.

The genus *Listeria* comprises facultative anaerobic Gram-positive rods and the genus includes both pathogenic and non-pathogenic species [1]. The foodborne pathogen *Listeria monocytogenes* is the species primarily responsible for the disease listeriosis in humans [1]. The main manifestations of the disease are septicaemia, abortion, and meningoencephalitis. Listeriosis primarily affects pregnant women, unborn children, newborns, the elderly, and adults with weakened immune systems [1]. In 2024, 3,041 invasive human cases of listeriosis were reported in EU, corresponding to a European Union notification rate of 0.69 cases per 100,000 population [2]. However, since 2021, the number of cases per 100,000 inhabitants per year has exceeded 1.0 in Sweden [3]. This high incidence is believed to be due to the higher consumption of smoked fish in the Scandinavian countries [4]. After the definitive report that *L. monocytogenes* is foodborne [1], much research has focused on investigating the sources and routes of this microorganism. Our experience of observing that one and the same food sample could contain several variants of *L. monocytogenes* [5] led us to investigate whether patients with listeriosis could also carry multiple types. We identified two unrelated strains in one patient [6]. We were also able to detect two closely related variants in blood cultures from each of two patients with listeriosis in 1999 and 2001, respectively [7]. Concerning the patient from 1999—an 86-year-old woman with suspected listeriosis—a blood sample (10 mL) was routinely taken at the hospital and inoculated into a BacT/ALERT FA bottle (Organon Teknika Corp., Durham, NC, USA). Following the diagnosis of listeriosis, the BacT/ALERT FA bottle was sent to our laboratory for further investigation. A volume of 0.1 mL of the contents was spread onto a 5% bovine blood agar plate and incubated at 37 °C for 24 h. Fifty colonies of *L. monocytogenes* were randomly picked from the diagnosed *L. monocytogenes* growth on the blood agar plate [7]. All isolates were frozen at − 70 °C in brain heart infusion (Merck, Darmstadt, Germany) containing 20% glycerol for later characterization. Each isolate was subsequently characterized by restriction enzyme analysis (*Asc* I and *Apa* I), followed by pulsed-field gel electrophoresis (PFGE). Two variants of *L. monocytogenes* were found among the 50 isolates: 37 isolates of variant 1, and 13 of variant 2. The variants displayed a two-band difference in DNA profile with both *Apa* I and *Asc* I (Fig. 1). Variant 2 included an insertion of about 100 kbp. One representative isolate of each of the two variants was serotyped and they were confirmed to share serovar 1/2a [6]. In 2008 in Canada, an outbreak of listeriosis occurred due to *L. monocytogenes* serovar 1/2a in ready-to-eat meat products. PFGE-typing of the epidemic isolates presented two similar, but distinct, *Asc* I patterns. Sequencing of isolates identified a prophage that accounted for the *Asc* I restriction pattern differences [8]. This result supports the theory that genetic differences between a closely related pair of isolates from the same outbreak mainly are due to changes in phage regions [9].

The aim of the present study was to determine whether the *L. monocytogenes* bacteria from the BacT/ALERT FA bottle from 1999 have survived, and if so, whether their PFGE profiles have changed. The above-mentioned blood culture bottle, stored in a refrigerator (5 ± 1 °C) for more than 25 years, was subjected in 2024 to a similar analysis to that performed in 1999. A volume of 0.1 mL of blood culture from the bottle and tenfold serial dilutions were surface-plated in 0.1 mL portions onto horse blood agar plates (Örebro University Hospital) and incubated at 37 °C for 24 h. One hundred colonies/isolates randomly picked from the above spreadings were frozen at -70 °C in brain heart infusion (Merck, Darmstadt, Germany) containing 20% glycerol for later characterization. Each isolate was characterized by PFGE and restricted with *Asc* I and *Apa* I enzymes according to the PulseNet standardized protocol [10], with the following modifications: The gel’s banding patterns were visualized with short-wave ultraviolet (312 nm) light and photographed with a Polaroid camera. The DNA profiles were analysed visually, and the position of each restriction fragment of each PFGE profile was sized against Lambda ladder PFG marker no. 340 S (New England BioLabs Inc., USA) to establish reference positions. PFGE types were established based on the number and distribution of all detectable fragments in a DNA restriction profile. All 100 selected isolates showed the same profile with the restriction enzyme *Asc* I as variant 1 did in 1999. Of those, fifty isolates showed the same profile with restriction enzyme *Apa* I. No isolate of variant 2—the variant with an insertion—was found with either *Asc* I or *Apa* I. After 25 years in a blood culture bottle, *L. monocytogenes* variant 1 was thus viable and countable, while variant 2, with the insertion, could not be found. In a review from 2001 it is stated that different variants of a main/dominant *L. monocytogenes* strain may be established during the passage and storage of the bacteria in the diagnostic laboratory, but it is also possible that they could constitute part of the infection process in the patient [11]. There is a possibility that variant 2 could be present but in very small quantities which can only be discovered if even more isolates are examined.

In an ice cream plant over seven years of sampling yielded *L. monocytogenes* serovar 1/2b, PFGE type II (defined by cleavage with *Asc* I, *Apa* I and *Sma*) in the environment, on the equipment, and in the ice cream [12]. PFGE type II was the dominant type among 11 *L. monocytogenes* serovar 1/2b PFGE types (26/41 isolates), and it survived in the ice cream plant for at least seven years. The authors stated that the *L. monocytogenes* strain of PFGE type II may have adapted to the production environment. Isolates of the other PFGE types (III–XII) were assumed to be variants of PFGE type II, since most of them differed by only one or two bands [12]. In addition, the PFGE types III–XII did not appear to persist in the production environment as well as *L. monocytogenes* PFGE type II, since isolates of other PFGE types were either single findings or appeared only twice, in the course of a single year [12].

In the present study, the BacT/ALERT FA bottle harbouring two variants of *L. monocytogenes* (1 and 2) may not have been a comfortable environment for variant 2, in a similar way to the situation for PFGE types III–XII in the ice cream plant [12]. In contrast to the ice cream plant environment, the BacT/ALERT FA bottle did not promote any noticeable change in the PFGE pattern of *L. monocytogenes* variant 1 over 25 years. The PFGE types II–XII from the ice cream plant were thought to have a clonal ancestor, as they differed very little from each other, and PFGE types III–XII are likely to be variants of PFGE type II, since most of them differed only by one or two bands [12].

The *L. monocytogenes* variants of a clonal ancestor that formed over the course of seven years in the ice cream plant [12] contrast with the strain (variant 1) in the blood culture bottle, where no change in DNA could be detected using PFGE. Our hypothesis is that, unlike the environment in the ice cream plant, the blood culture bottle was a closed compartment in which the *L. monocytogenes* culture was not exposed to any competition from other microorganisms. It should be kept in mind that the different variants of *L. monocytogenes* in the ice cream plant that were isolated for seven years may have been introduced from outside [12]. However, this is less likely, as the variants all have closely related PFGE profiles compared to the clonal ancestor. In a study from 2021 it is reported that bacterial cells can survive for months to years without the addition of nutrients, and must therefore rely on cellular detritus that they recycle [13]. The authors maintained a single strain of *Escherichia coli* cells for 1200 days in a batch culture without the addition of fresh medium. *L. monocytogenes* can apparently survive for a long time in a nutrient-poor environment, and this must be taken into account by the food industry. Persistence in clinical samples has rarely been reported; however, several authors report persistence of *L. monocytogenes* in the food industry, with bacteria ending up in final products. A *L. monocytogenes* strain persisted for 12 years (1988–2000) in a food-processing plant in Texas causing listeriosis due to contaminated turkey meat [14] and *L. monocytogenes* subtypes were repeatedly found in a smoked fish processing facility [15]. PFGE analysis of 141 isolates supported persistence for up to 11 years. In a review the persistence times for *L. monocytogenes* in different food processing plants are listed from one month to eight years [16]. The capacity of long-term survival of *L. monocytogenes* in nutrient poor environment, such as soil, is illustrated in several papers [17]. In a review from 2026 the agricultural systems, soils and livestock are proposed to act as chronic reservoirs [18].

After more than 25 years in a refrigerator, *L. monocytogenes* bacteria from an invasive case of listeriosis inoculated into a BacT/ALERT FA bottle was isolated in pure culture. *L. monocytogenes* can apparently survive for a long time in a nutrient-poor environment, and this must be taken into account by the food industry. Persistence in clinical samples has rarely been reported; however, several authors report persistence of *L. monocytogenes* in the food industry, with bacteria ending up in final products. The present study highlights the potential of retrospective study on blood culture.

## Data Availability

Not applicable.
